# Changes in weekend and weekday care quality of emergency medical admissions to 20 hospitals in England during implementation of the 7-day services national health policy

**DOI:** 10.1136/bmjqs-2020-011165

**Published:** 2020-10-28

**Authors:** Julian Bion, Cassie Aldridge, Alan J Girling, Gavin Rudge, Jianxia Sun, Carolyn Tarrant, Elizabeth Sutton, Janet Willars, Chris Beet, Amunpreet Boyal, Peter Rees, Chris Roseveare, Mark Temple, Samuel Ian Watson, Yen-Fu Chen, Mike Clancy, Louise Rowan, Joanne Lord, Russell Mannion, Timothy Hofer, Richard Lilford

**Affiliations:** 1 Department of Intensive Care Medicine, College of Medical and Dental Sciences, The University of Birmingham, Birmingham, UK; 2 Department of Intensive Care Medicine, Institute of Clinical Sciences, University of Birmingham, Birmingham, UK; 3 Department of Health Informatics, University Hospitals Birmingham NHS Foundation Trust, Birmingham, UK; 4 Department of Health Sciences, University of Leicester, Leicester, UK; 5 Department of Intensive Care, University Hospitals Coventry and Warwickshire NHS Trust, Coventry, UK; 6 Department of Research & Development, University Hospitals Birmingham NHS Foundation Trust, Birmingham, UK; 7 Academy of Medical Royal Colleges, London, UK; 8 Department of Gastroenterology, Southern Health NHS Foundation Trust, Southampton, UK; 9 Renal Medicine, University Hospitals Birmingham NHS Foundation Trust, Birmingham, UK; 10 Division of Health Sciences, Medical School, University of Warwick, Coventry, Warwickshire, UK; 11 Emergency Medicine, Southampton University Hospitals NHS Trust, Southampton, UK; 12 University of Southampton, Southampton, Hampshire, UK; 13 Health Services Management Centre, University of Birmingham, Birmingham, UK; 14 Department of Medicine, University of Michigan Medical School, Ann Arbor, Michigan, USA; 15 Department of Public Health, Epidemiology & Biostatistics, University of Birmingham, Birmingham, UK

**Keywords:** emergency department, health policy, health services research, medical error, measurement/epidemiology, quality improvement

## Abstract

**Background:**

In 2013, the English National Health Service launched the policy of 7-day services to improve care quality and outcomes for weekend emergency admissions.

**Aims:**

To determine whether the quality of care of emergency medical admissions is worse at weekends, and whether this has changed during implementation of 7-day services.

**Methods:**

Using data from 20 acute hospital Trusts in England, we performed randomly selected structured case record reviews of patients admitted to hospital as emergencies at weekends and on weekdays between financial years 2012–2013 and 2016–2017. Senior doctor (‘specialist’) involvement was determined from annual point prevalence surveys. The primary outcome was the rate of clinical errors. Secondary outcomes included error-related adverse event rates, global quality of care and four indicators of good practice.

**Results:**

Seventy-nine clinical reviewers reviewed 4000 admissions, 800 in duplicate. Errors, adverse events and care quality were not significantly different between weekend and weekday admissions, but all improved significantly between epochs, particularly errors most likely influenced by doctors (clinical assessment, diagnosis, treatment, prescribing and communication): error rate OR 0.78; 95% CI 0.70 to 0.87; adverse event OR 0.48, 95% CI 0.33 to 0.69; care quality OR 0.78, 95% CI 0.70 to 0.87; all adjusted for age, sex and ethnicity. Postadmission in-hospital care processes improved between epochs and were better for weekend admissions (vital signs with National Early Warning Score and timely specialist review). Preadmission processes in the community were suboptimal at weekends and deteriorated between epochs (fewer family doctor referrals, more patients with chronic disease or palliative care designation).

**Conclusions and implications:**

Hospital care quality of emergency medical admissions is not worse at weekends and has improved during implementation of the 7-day services policy. Causal pathways for the weekend effect may extend into the prehospital setting.

## Introduction

In 2013, National Health Service England launched the 7-day services programme^1^ ‘*designed to ensure patients that are admitted as an emergency, receive high quality consistent care, whatever day they enter hospital*’.^2^ The programme consisted of 10 service delivery standards of which six involved increasing consultant involvement in frontline care. The stimulus for this policy derived in part from the perception that the higher mortality associated with weekend admission to hospital was attributable to the absence of senior medical staff at weekends.^3 4^ This theory was first proposed by Bell and Redelmeier^5^ in 2001, but in the accompanying editorial,^6^ Halm and Chassin^6^ observed that ‘*Disentangling the potential causal pathways would require painstaking detective work*’. Since then more than 600 studies of the weekend effect have been published; our group has recently undertaken a meta-ananlysis of 68 studies involving 640 million general unselected emergency and elective weekend admissions to hospital, with a pooled excess relative risk of mortality of 16% for weekend admissions.^7^ However, few studies have conducted the ‘painstaking detective work’ to elucidate the potential causal pathways.

Recent studies suggest multifactorial causes for the weekend effect. Weekend admissions are sicker,^8–11^ and there is also a denominator contribution from fewer patients being admitted at weekends despite a similar emergency department (ED) attendance rate.^8 12^ A cross-sectional analysis of hospitals in England^13^ found a marked reduction in specialist (consultant) intensity at weekends but no relationship between specialist intensity and risk of death for weekend emergency admissions. Moreover, there does not appear to be a relationship between weekend admission mortality and the adoption of 7-day service standards.^14^ In theory, reduced weekend staffing and resources should affect all hospitalised patients not just those newly admitted, but studies have shown a lower mortality rate among already-hospitalised patients at weekends compared with weekdays.^3 15^

None of these studies assessed hospital quality of care, the putative mediating variable for increased risk of death for weekend admissions. Previous studies of quality of healthcare have shown a tendency towards improvement over time^16 17^ but did not examine weekend:weekday differences. More recently a study of stroke care across England has shown improvements in outcomes for weekend admissions over time, unrelated to centralisation of services.^18^ We therefore examined error and associated adverse event rates among 4000 patients admitted as emergencies at weekends and on weekdays to 20 hospital Trusts in England during two epochs representing the preimplementation and postimplementation phases of the roll-out of 7-day services. We also attempt to explicate causal links by studying patient admission pathways and case mix, as part of the High-intensity Specialist Led Acute Care (HiSLAC) project^19^ funded by the National Institute for Health Research, Health Services and Delivery Research (HS&DR) programme.

## Aims

To determine whether the quality of hospital care of patients admitted as medical emergencies is worse at weekends and whether care quality has changed between epochs during the implementation of 7-day services.

## Methods

We compared error rates, quality of care and patient admission pathways between weekend and weekday admissions in hospitals with higher and lower specialist intensities at weekends and between epochs. We recapitulate briefly here the methodology that has been described in detail previously.^20^

### Selection of hospital Trusts

We invited 20 of the 115 acute hospital Trusts in England participating in the HiSLAC project^13^ to take part. Trusts were classified first into quintiles of size (acute beds) and then four were selected from within each quintile, two with the highest and two with the lowest Sunday specialist intensity (2014 data) (online supplemental table 1). Data on specialist intensity (hours of consultant time per 10 emergency admissions) were derived from the HiSLAC national point prevalence survey conducted annually on a Sunday and a Wednesday in June between 2014 and 2018^20^ (data on specialist intensity for the 5 years is in press, *Health Services and Delivery Research Journal* 2020). Following an on-site initiation visit, each Trust provided an anonymised and hash-encrypted Patient Administration System (PAS) dataset for all admissions during two epochs, financial year 1 April 2012–31 March 2013 and 1 April 2016–31 March 2017.

10.1136/bmjqs-2020-011165.supp1 Supplementary data

**Table 1:** Number of errors, error-related adverse events and process indicators, by weekend–weekday admission and epoch

	Both epochs			Epoch 1			Epoch 2		
	Total	W/E	W/D	Total	W/E	W/D	Total	W/E	W/D
	n (%)	n (%)	n (%)	n (%)	n (%)	n (%)	n (%)	n (%)	n (%)
Total	**4000**	**2000**	**2000**	**2000**	**1000**	**1000**	**2000**	**1000**	**1000**
Total number of errors	1618	803	815	914	440	474	704	363	341
Number of errors									
None	2970 (74.3)	1486 (74.3)	1484 (74.2)	1447 (72.4)	723 (72.3)	724 (72.4)	1523 (76.2)	763 (76.3)	760 (76.0)
Any error	996 (24.9)	497 (24.9)	499 (25.0)	541 (27.1)	269 (26.9)	272 (27.2)	455 (22.8)	228 (22.8)	227 (22.7)
1	645 (16.1)	328 (16.4)	317 (15.9)	338 (16.9)	178 (17.8)	160(16)	307 (15.4)	150 (15.0)	157 (15.7)
2	208 (5.2)	100 (5.0)	108 (5.4)	121 (6.1)	53 (5.3)	68 (6.8)	87 (4.4)	47 (4.7)	40 (4.0)
3	83 (2.1)	36 (1.8)	47 (2.4)	43 (2.2)	18 (1.8)	25 (2.5)	40 (2.0)	18 (1.8)	22 (2.2)
4	31 (0.8)	17 (0.9)	14 (0.7)	22 (1.1)	12 (1.2)	10 (1.0)	9 (0.5)	5 (0.5)	4 (0.4)
5 or more	29 (0.7)	16 (0.8)	13 (0.7)	17 (0.9)	8 (0.8)	9 (0.9)	12 (0.6)	8 (0.8)	4 (0.4)
Missing	34 (0.9)	17 (0.9)	17 (0.9)	12 (0.6)	8 (0.8)	4 (0.4)	22 (1.1)	9 (0.9)	13 (1.3)
Mean number of errors per patient admission*	0.408	0.405	0.411	0.460	0.444	0.476	0.356	0.366	0.345
Patients with one or more adverse events	103	49	54	68	31	37	35	18	17
Process indicators									
Location not appropriate	118 (3.0)	59 (3.0)	59 (3.0)	61 (3.1)	29 (2.9)	32 (3.2)	57 (2.9)	30 (3.0)	27 (2.7)
Incomplete vital signs	1280 (32.0)	596 (29.8)	684 (34.2)	597 (29.9)	280 (28.0)	317 (31.7)	683 (34.2)	316 (31.6)	367 (36.7)
NEWS not recorded	2060 (51.5)	982 (49.1)	1078 (53.9)	1090 (54.5)	521 (52.1)	569 (56.9)	970 (48.5)	461 (46.1)	509 (50.9)
No specialist review <14 hours documented	2811 (70.3)	1393 (69.7)	1418 (70.9)	1448 (72.4)	727 (72.7)	721 (72.1)	1363 (68.2)	666 (66.6)	697 (69.7)

*Calculated using 'total number of errors ÷ (all records excluding errors missing)'.

NEWS, National Early Warning Score; W/D, weekday; W/E, weekend.

### Case record review

We based our approach to obtaining case records on the method used for the evaluation of the Safer Patient Initiative.^21^ We chose not to confine the study to mortality reviews in order to avoid endogenous selection bias (from the outcome influencing the sample) and to ensure that the study population was representative. We focused the study on non-operative emergency medical admissions, that is, patients who were not admitted for surgery, using a code to identify non-surgical procedures. Following submission and data cleaning, from each Trust’s PAS datasets, we randomly selected 200 admissions, 100 from each epoch, each with 50 weekend and 50 weekday admissions, a total of 4000 unique admissions. Trusts were reimbursed £600 for staff to copy and scan the case records, masking patient identifiers (name, address, age and postcode); records were censored for lengths of stay exceeding 7 days. All available documents relating to the first 7 days of the admission were included: ambulance and ED records, physician and nursing entries, correspondence and reports of laboratory and radiological tests. Records of previous admissions or readmissions were not included, only the index admission. Radiological reports were included but not the images. Record completeness was assured using a checklist. Files were transferred using a file share program to a central repository at the University of Birmingham and checked for anonymisation. Complete records were uploaded to REDCap^22^ and allocated randomly to the reviewers.

The 79 case record reviewers were consultants (attendings) and senior registrars (senior residents) in acute medical specialities. Reviewers attended one of three centralised half-day practical training sessions in case record reviewing (data identification, error typology, adverse events, care quality and bias). Reviewers accessed the password-protected case records online independently in their own time. Progress was monitored every 2 weeks, with group reminders and personal contacts if required. An honorarium of £10 per completed review was paid at the end of the project.

### Patient admission pathways and care processes

Reviewers identifed from the case records the patients’ preadmission and postadmission pathways including how patients arrived at the hospital (referral from family doctor, emergency ambulance and self-presentation), vital signs documentation and calculation of the National Early Warning Score (NEWS), initial and subsequent location following admission, timeliness of specialist review and palliative care decisions. Case mix was derived from PAS data.

### Assessment of errors, adverse events, preventability and global assessment of care quality

We employed structured judgement review^23–25^ to identify and characterise errors and associated adverse events. This is the recommended approach for national mortality reviews in the UK, facilitating a degree of standardisation in decision making while still permitting individual judgement. Reviewers were not blinded to dates because of the requirement to determine timeliness of specialist reviews. Error typologies were based on those used by Hogan *et al*.^23^ Reviewers then gave a free-text description of the error; more than one typology could be chosen per error. Error-related adverse events were graded for preventability using a six-point scale from ‘virtually no evidence for preventabiity’ to ‘virtually certain evidence’.^26^ Error-related adverse events (corresponding to ‘preventable adverse events’) distinguish adverse events preceded by an error from those attributable to the underlying disease(s). Reviewers gave each case a global assessment of care quality (‘To what extent did this patient receive best practice care?’) using a five-point scale from ‘completely’ to ‘not at all’. The data collection fields are provided in the published protocol.^20^

### Patient and public involvement (PPI)

Patients and the public have been involved in three ways. First, a PPI representative (PR) has been a full collaborator in the project from inception, contributing to metric development and interpretation of results. Second, a PPI representative (PS) has been a full and active member of the Oversight and Governance Committee. Third, patients and relatives contributed to the information gleaned by the ethnographers during their site visits to the 20 Trusts (data not presented here).

### Statistical analysis

The primary outcome was the rate of clinical errors among emergency admissions. Secondary outcomes included: error-related adverse event rates, global quality of care assessments and four explicit indicators of good practice (appropriate initial treatment location, completeness of vital signs and NEWS reporting and timeliness of specialist review, ie, within 14 hours of admission).

For the main analysis, each of the 4000 case records was reviewed by one of 79 reviewers. In addition, 800 records (40 from each trust, of which 20 from each epoch) were selected for a second review by a randomly chosen reviewer to assess inter-reviewer reliability. Reviewer reliability coefficients were computed from these repeat reviews. Intraclass correlation coefficients (with class=case record) were used for errors and adverse events and a (linearly) weighted kappa coefficient for the 5-point quality of care Likert scale. The reliability of aggregated assessments (within Trusts and epochs) was estimated using the Spearman-Brown formula.^27^

The outcomes were analysed using mixed effects generalised linear models. Negative binomial models were used for numbers of errors, logistic models for adverse events and process indicators and an ordinal logistic model for the quality of care Likert scale. In all models, fixed effects were fitted for hospital Trust, day of week (weekend/weekday) and time-epoch; random effects were fitted for reviewers. All models were adjusted for patient age (using restricted cubic splines with five knots), sex and ethnicity (Caucasian, non-Caucasian and missing). Changes in the weekend effects over time were captured by adding day by epoch interaction terms to the mixed effects models. Trust-level effects were extracted for correlation analysis with estimates of specialist involvement (specialist hours per 10 emergency admissions) from the point prevalence survey. Peason’s χ^2^ test was used to compare the difference between epochs, weekend versus weekday, for the preadmission data.

### Ethics

Informed consent was not required for accessing anonymised patient records.

## Results

### Demographics

Four thousand case records were retrieved. The characteristics of the randomly selected study population were representative of the hospital admitted population in England (online supplemental table 2). Median length of hospital stay was 2 days.

**Table 2:** Analysis of error rates, adverse events, quality of care and process indicators between epochs, between day of admission and between day of admission between epochs

	Epochs (epoch2:epoch1)		Day of admission (weekend:weekday)		Weekend:weekday	
					Between epochs	
	RR (OR)* (confidence limits)	P value	RR (OR)* (confidence limits)	P value	RR (OR)* (confidence limits)	P value
Errors						
Assessment, investigation or diagnosis	0.71 (0.61 to 0.83)		0.93 (0.79 to 1.10)		1.14 (0.77 to 1.69)	
Treatment and management	0.74 (0.63 to 0.87)		0.97 (0.83 to 1.13)		1.18 (0.80 to 1.73)	
Communication	0.84 (0.66 to 1.07)		1.08 (0.87 to 1.34)		1.44 (0.93 to 2.23)	
Medication	0.65 (0.52 to 0.81)		0.92 (0.72 to 1.19)		0.86 (0.50 to 1.47)	
Monitoring	0.78 (0.55 to 1.11)		0.94 (0.70 to 1.27)		0.89 (0.36 to 2.21)	
Resuscitation	0.82 (0.37 to 1.81)		2.61 (1.15 to 5.91)		1.14 (0.16 to 8.03)	
Infection	2.35 (0.71 to 7.76)		0.73 (0.24 to 2.26)		1.23 (0.13 to 11.5)	
Invasive procedures	0.46 (0.20 to 1.04)		1.63 (0.70 to 3.77)		1.15 (0.21 to 6.27)	
Other	0.52 (0.30 to 0.92)		1.21 (0.78 to 1.86)		1.20 (0.44 to 3.29)	
All errors	0.78 (0.70 to 0.87)	<0.0001	0.96 (0.86 to 1.07)	0.4922	1.14 (0.91 to 1.45)	0.2566
Adverse events	0.48 (0.33 to 0.69)	0.0001	0.89 (0.57 to 1.38)	0.5991	1.29 (0.54 to 3.08)	0.5663
Global quality of care	0.78 (0.70 to 0.87)	<0.0001	0.98 (0.86 to 1.10)	0.6904	1.03 (0.81 to 1.31)	0.7973
Process indicators						
Location not appropriate	0.91 (0.64 to 1.30)	0.6170	1.00 (0.71 to 1.42)	0.9961	1.16 (0.54 to 2.46)	0.7051
Incomplete vital signs	1.25 (1.07 to 1.47)	0.0056	0.80 (0.71 to 0.91)	0.0009	0.99 (0.74 to 1.32)	0.9267
NEWS not recorded	0.75 (0.65 to 0.88)	0.0003	0.81 (0.71 to 0.93)	0.0026	1.06 (0.79 to 1.43)	0.6805
Specialist review <14 hours not documented	0.82 (0.72 to 0.93)	0.0026	0.95 (0.81 to 1.11)	0.5142	0.86 (0.68 to 1.09)	0.2194

Adjusted for age, sex, ethnicity and hospital trust.

*Rate ratios for errors (from mixed effects negative binomial models); ORs for adverse events and process indicators (from mixed effects binary logistic models); proportional ORs for global quality of care (from mixed effects ordinal logistic regression).

OR, odds ratio; RR, rate ratio.

### Case records and reviews

**Figure 1:**
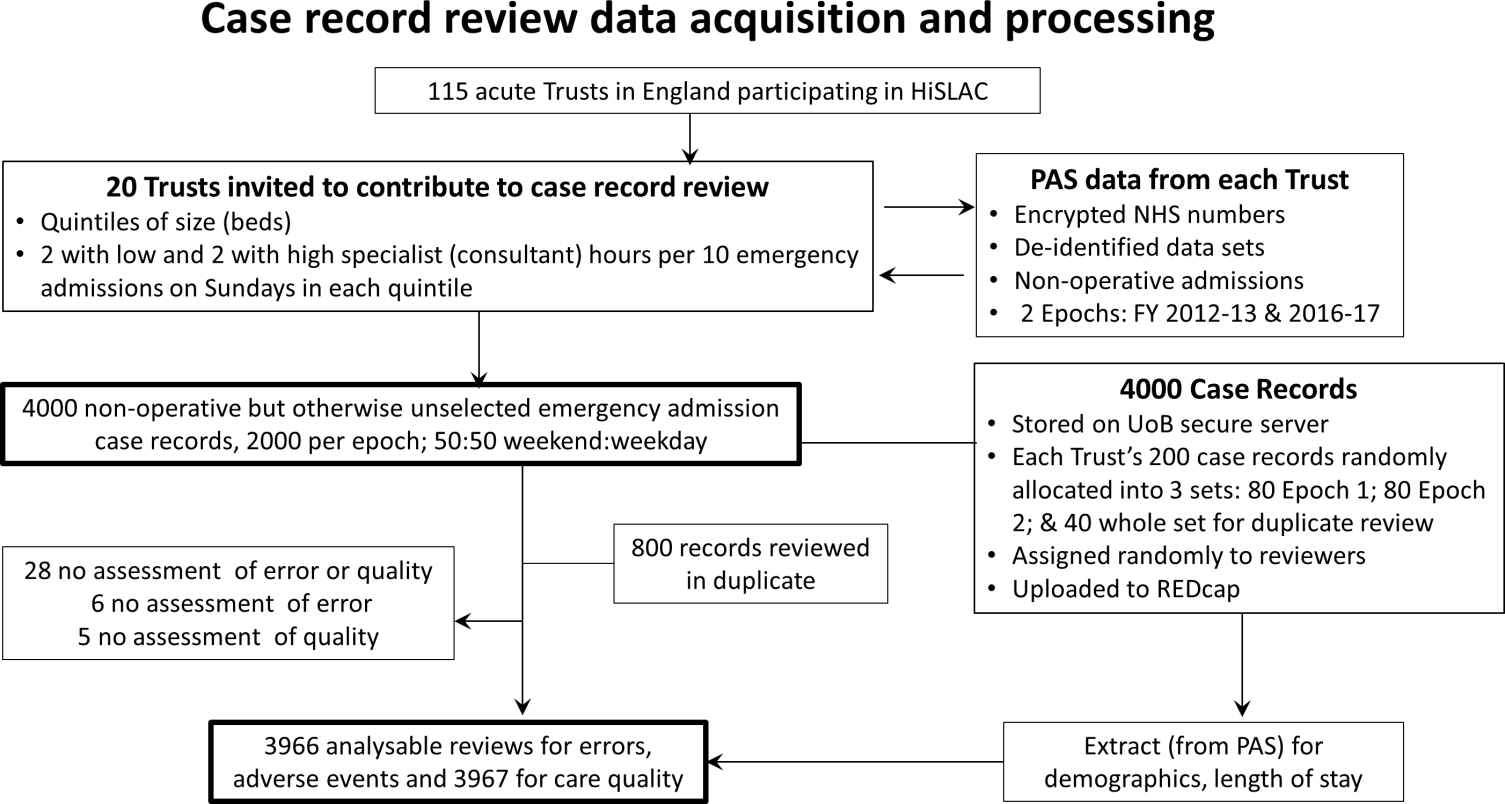
Case record review data acquisition and processing. HiSLAC, High-intensity Specialist Led Acute Care; NHS, National Health Service; PAS, Patient Administration System; UoB, University of Birmingham.

Seventy-nine reviewers participated; the mean number of reviews per reviewer was 61 (20–69), with 800 records reviewed in duplicate. Reviewers felt unable to provide an assessment of errors and global assessment of care quality in 28 (insufficient documentation), of error alone in 6 and of quality alone in 5. Of the 1600 duplicate reviews, 1584 could be used for inter-reviewer reliability of assessment of error and 1586 for care quality (figure 1).

### Admission pathways

Data extracted by the reviewers on the preadmission pathway are summarised in online supplemental table 3a and the postadmission pathway in online supplemental table 3b.

**Table 3:** Inter-reviewer reliability

Error category and global quality	All reviews (n=4763)	Errors per review			Repeat reviews (n=1584 = 2 × 792 reviews in total)		
	Errors	Mean	SD	Max.	Errors	Individual-level reliability*	Trust-level reliability†
Assessment	903	0.19	0.58	12	272	0.003	0.138
Treatment	824	0.17	0.54	10	265	0.131	0.883
Communication	442	0.09	0.44	14	140	0.058	0.753
Medication	380	0.08	0.34	8	129	0.072	0.794
Monitoring	138	0.03	0.19	4	47		
Resuscitation	38	0.01	0.10	2	12		
Infection	26	0.01	0.08	2	11		
Invasive	25	0.01	0.08	2	5		
Other	75	0.02	0.13	2	25		
All errors	1909	0.40	0.92	15	606	0.026	0.568
Global QoC						0.105	0.854

*Omitting categories with fewer than 50 errors reported among the repeat reviews.

†Computed from the Spearman-Brown formula with 50 case notes per trust.

QoC, Quality of Care.

Preadmission pathways (online supplemental table 3a): the majority of patients were admitted from home. Weekend admissions were more likely to be dependent on others for activities of daily living (weekend 11.0% vs weekday 8.3%, p=0.0038) and to reach hospital by emergency ambulance (51.6% vs 42.1%, p<0.0001), less likely to have been referred by a general practitioner (8.2% vs 19.8%, p<0.0001) and less likely to be admitted directly to an acute ward bypassing the ED (8.0% vs 14.6%, p<0.0001). These weekend–weekday differences were more marked for the second epoch than the first. Weekend admissions were more likely to include patients in whom a palliative care decision was already in place, or was applied at the time of admission, or in the opinion of the reviewer should have been made (17.1% vs 14.1%, p=0.0089), with a marked increase between epochs (13.2% vs 18.1%, p<0.0001). Fewer weekend than weekday admissions were considered definitely or possibly avoidable by the reviewers (24.3% vs 28.4%, p=0.0032).

### Postadmission errors, error-related adverse events, global quality of care and care processes

Errors: of the 4000 case records (equally divided between weekend and weekday admission), 3966 could be assessed for errors and 3967 for care quality. One or more errors in care were identified in 996 records: 1618 errors were identified in total. Single errors were identified for 16.1% of reviews and two or more for 8.8% (table 1). The most frequent category of error was ‘clinical assessment, investigation or diagnosis’ (31.9%) followed by ‘treatment and management’ (29.1%), ‘communication’ (15.3%) and ‘medication’ (13.2%) (online supplemental table 4).

Adverse events: reviewers identified 128 adverse events in 103 patients (2.6%). Ninety-one adverse events were judged to have a >50% chance of being preventable (online supplemental table 4).

Global quality assessment: reviewers considered that best practice care had been provided completely in 1579 (39.5%) of cases, substantially in 1659 (41.5%), partially in 623 (15. 6%), very little in 83 (2.1%) and not at all in 23 (0.8%) (online supplemental table 4).

Care processes (tables 1 and 2 and online supplemental table 3b): the initial location for admission (usually the acute medical unit) was considered appropriate for most admissions regardless of the day of admission. Vital signs were incomplete for 32.0% of admissions, calculation of NEWS was absent in 51.5% and specialist review within 14 hours was not formally documented in 70.3%.

### Weekend:weekday admission differences

Of the 1618 identified errors, 803 were in weekend admissions and 815 in weekday admissions (table 1). The overall error rate per case was similar for weekend (0.405) and weekday (0.411) admissions (adjusted rate ratio 0.96; 95% CI 0.86 to 1.07; p=0.4922) (table 2, middle column). Similarly, there was little difference in adverse event rates or global care quality between weekend and weekday admissions. Documentation of vital signs and calculation of a NEWS were more complete for weekend admissions (50.9% vs 46.1%) (online supplemental table 3b), with consequential improvements in two of the process indicators (adjusted ORs 0.80; 95% CI 0.71 to 0.91 and 0.81; 95% CI 0.71 to 93). Initial specialist review within the first 14 hours following admission (combining ‘documented’ with ‘probable’ specialist reviews) indicated that this had occurred in 1189 (29.7%) of cases overall, 30.4% for weekend admissions and 29.1% for weekday (online supplemental file 1).

### Temporal trends between epochs (2012–2013 and 2016–2017)

In contrast to weekend:weekday admission comparisons, the overall error rate between epochs reduced significantly (adjusted rate ratio 0.78; 95% CI 0.70 to 0.87; p<0.0001), and this was reflected to some extent in every error category save that of ‘Infection’ (table 2). A significant reduction was also observed between epochs in error-related adverse events; 103 patients suffered 128 adverse events, 68 in epoch 1 and 35 in epoch 2 (adjusted OR 0.48, 95% CI 0.33 to 0.69, p=0.0001) (tables 1 and 2). There was some improvement in care quality (adjusted OR 0.78, 95% CI 0.70 to 0.87, p=0.0001, table 2): for instance, the proportion of reviews attracting the two highest care quality assessments (‘completely’ and ‘substantially’) rose from 79.4% in Epoch 1 to 82.5% in Epoch 2 (online supplemental table 4). There was no evidence for temporal change in weekend:weekday differences over time for any outcome measure (table 2, final column).

The indicators for calculation of NEWS (adjusted OR 0.75; 95% CI 0.65 to 0.85) and timely consultant review (adjusted OR 0.82; 95% CI 0.72 to 0.92) improved over time. Indeed, the documentation of vital signs and calculation of the NEWS changed from 47.9% to 53.9% at weekends and from 43.1% to 49.1% on weekdays (online supplemental table 3b). However, the proportion of cases in which vital signs were absent or incomplete increased between epochs (adjusted OR 1.25; 95% CI 1.07 to 1.47). Specialist review within 14 hours of admission was documented in the case record in 22.6% (weekend) and 24.8% (weekday) of case reviews for epoch 1, increasing in epoch 2 more markedly for weekend than weekday admissions (30.3% vs 27.2%, respectively). Combining ‘documented’ with ‘probable’ specialist review within 14 hours showed no difference between weekends and weekdays but an improvement between epochs (weekends 27.3%–33.4%; weekdays 27.9%–30.3%).

### Inter-reviewer differences

There was substantial variation between reviewers in error identification rates (online supplemental figure 1). Repeat reviewer assessments were available for 792 case records for error and 793 for global quality. Reviewer reliability coefficients were generally low (table 3). However, the study does not aim to establish the quality of care for any particular patient but is concerned with aggregate data within individual Trusts; that is, the 50 case notes representing each Trust in a particular epoch for weekend or weekday admissions. The reliability of such aggregates estimated using the Spearman-Brown formula is much higher, as shown in table 3.

### Trust-level aggregate measures

**Figure 2:**
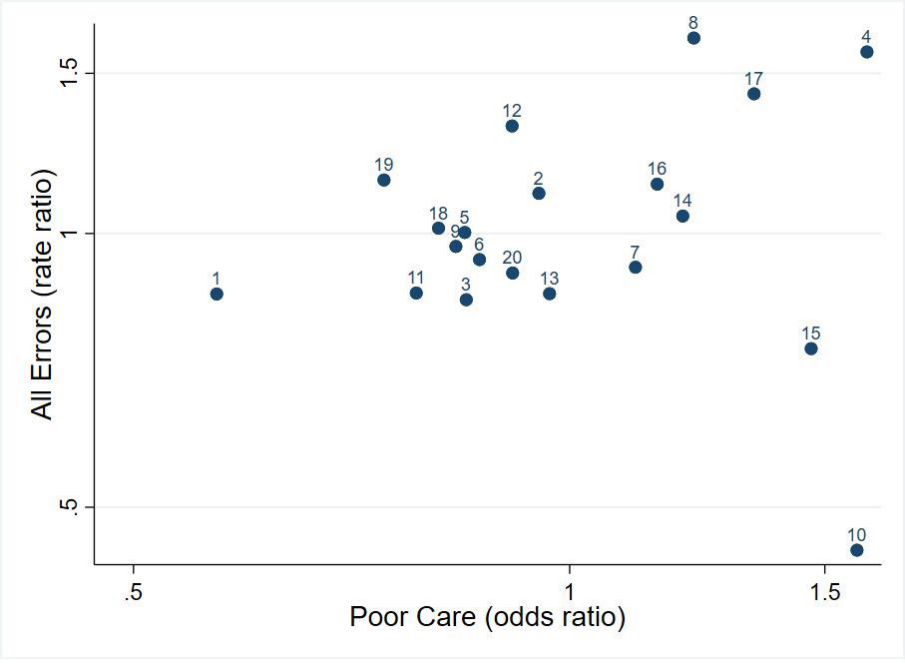
Error and global care quality. Rate-ratio for the presence of all errors and the OR for (worse) quality of case are plotted on a logarithmic scale, with a value of 1 corresponding to the average trust.

Error and global quality: the relationship between error rates and global care quality at Trust level is represented in figure 2. Estimates of the Trust level (for ‘All Errors’ and ‘Global Quality’ of care) obtained from the models reported in table 2 are plotted against one another. The estimates came with (average) SEs of 0.15 from the error model and 0.16 from the quality of care model. Since the SD of the actual estimates was 0.28 for both errors and quality of care, this means that about 70% (≈ 1 − (0.15/0.28)^2^) of the variation in the figure is due to genuine differences between trusts. Nevertheless, the overall correlation is not convincing (r=0.168, p=0.478); indeed, Trusts 10 and 15 recorded the lowest error rates but were among the bottom three for care quality assessments.

### The weekend effect and specialist intensity

As we found no relationship between errors and day of admission, but a clear reduction in error rates between epochs, we undertook exploratory analyses related to specialist intensity. Specialist intensity was both a criterion for selecting the 20 Trusts (10 low and 10 higher weekend intensity) and a secondary outcome measure in terms of change over time. We therefore tried to determine if the secular change was associated with an overall improvement in specialist intensity—the rising tide phenomenon.

**Figure 3:**
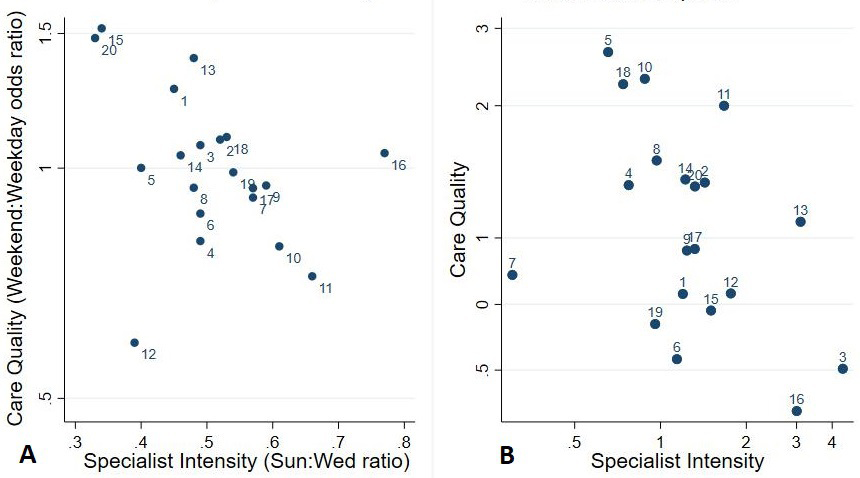
Trust-level weekend effects for quality of care and specialist intensity: (A) pooled over both epochs and all PPS time points. (B) Relative changes between two epochs. (A) a value above 1 indicates that the gap between weekend and weekday care is worse than average. (B) a value below 1 indicates that the gap between weekend and weekday care has improved over time. PPS, Point Prevalence Survey.

The specialist intensity point prevalence surveys were conducted over a 5-year period (2013/2014–2017/2018), which spanned the epochs of the case note review. Specialist intensity was defined as the number of dedicated specialist hours per 10 emergency admissions. The data were used to estimate the weekend:weekday intensity ratio for each trust. The average over the 20 trusts of the intensity ratio obtained from the 5 years of survey data was 0.51 (SD 0.11) reflecting a much lower specialist attendance at weekends. The 7-day initiative is predicated on the assumption that the equalisation of hospital services across the week would lead to an improvement in the quality of weekend care relative to weekdays. This might mean: (A) that Trusts with relatively higher weekend:weekday intensity ratios tend to deliver more equal standards of care across the week and/or (B) that Trusts where the intensity ratio has increased between the two epochs will show a corresponding improvement in weekend care relative to weekdays. These possibilities are examined in figure 3A, B.

In figure 3A, Trust-level weekend effects for quality of care (ie, weekend:weekday ratios pooled over both epochs) are plotted against the corresponding 5-year intensity ratios. The correlation is negative, as expected, indicating that Trusts with a larger weekend:weekday difference in specialist staffing also have a larger difference in care quality, but the correlation is not formally significant (r=−0.433, p=0.057).

In figure 3B, we examine changes over time. The intensity ratios from 2013 to 14 (mean 0.47, SD 0.18) were taken as representative of epoch 1, and those from 2016 to 17 (mean 0.58, SD 0.27) as representative of epoch 2. In this way, a ratio of weekend effects between epochs can be calculated for each Trust, both for global quality of care and for specialist intensity. The resulting plot yields an almost identical correlation to figure 3A (r=−0.428, p=0.060), consistent with the interpretation that the gaps between weekend and weekday care quality and specialist intensity have both tended to narrow over time.

A similar analysis using Trust-level weekend effects for the presence of error in place of poor care quality (figure not shown) produced a somewhat smaller negative correlation (r=−0.34, p=0.149).

## Discussion

The national 7-day services policy^1^ was intended to improve quality of care for patients admitted to hospital as emergencies at weekends by requiring hospital Trusts to increase specialist (consultant) input and services to a level similar to that of weekdays. However, there was little evidence that care quality in hospital was actually worse at weekends or that increasing specialist intensity would improve outcomes. In this two-epoch study of 4000 case records across 20 Trusts, we find no support for the concept that the quality of in-hospital medical care of patients undergoing emergency admission is lower for patients admitted at weekends compared with weekdays. If there is a signal, it would suggest some slight advantage for weekend admissions. However, our study provides strong evidence of a temporal trend towards improved hospital care between epochs: there was a statistically significant improvement in error rates, error-related adverse events and global quality of care assessments. These findings triangulate well with improvements in in-hospital processes of care between epochs (initial specialist review; recording NEWS). They are also aligned with the ‘rising tide’ phenomenon^28^ of secular improvements in care processes reported previously.^16^ The error-related adverse event rate of 2.6% in our set of relatively short-stay admissions is consistent with a recent systematic review reporting a median prevalence of 5% (IQR 3%–9%) for preventable adverse events across 70 studies between 2000 and 2018.^29^

The 7-day services initiative^1^ may have contributed to the reduction in error rates between epochs, particularly those most influenced by doctors—assessment, diagnosis, treatment, prescribing and communication—by promoting timely specialist reviews across all days of the week. There may be a moderate relationship between specialist intensity and judgements of care quality (figure 3A), and a trend for Trusts, which narrow the Sunday:Wednesday specialist intensity gap between epochs 1 and 2 also to reduce the weekend:weekday global care quality gap between epochs (figure 3B), though neither achieves conventional statistical significance (alpha 5%) at this sample size. Other factors that might have contributed to improved care over time include increased input from allied health professionals,^30^ or the introduction of electronic prescribing and patient records, though benefits of digitisation compared with paper records remain uncertain.^31–33^ It is possible that the reduction in raw vital signs recording between epochs with a concurrent increase in complete vital signs with NEWS calculation could represent a transition to electronic recording and automated NEWS calculation.

It should also be noted that the absence of a weekend effect for care quality in hospital does not mean that care quality overall is satisfactory: there is scope for improvement in documenting vital signs and in timely specialist review across all days of the week. Even in the second epoch, a consultant review was documented in only half the case records and within 14 hours of admission in only one-third. Further work is needed to determine whether the absence of consultant review is associated with a decrement in care quality.

The prehospital data were included as part of the demographic descriptors and are presented here for hypothesis generation. Prehospital processes appear to contrast markedly with the in-hospital postadmission data. Patients admitted at weekends were more likely to be physically dependent, to have a palliative care decision in place and to have arrived by ambulance into the ED and much less likely to have been referred by a general practitioner in the community. All these indicators were more marked in the second epoch than the first. These findings are consistent with other studies showing that weekend admissions from the community are sicker than those admitted on weekdays^8 9^ and that there are fewer GP referrals at weekends.^8 12^ As these changes have occurred at the same time as a reduction in social care funding despite increasing demand,^34^ these findings suggest the possibility that at weekends there is a decrement in community care of vulnerable patients and that this has deteriorated with time.

### Limitations and mitigation

Case record review is the most common method used in population-based assessments of adverse events and hospital quality of care but lacks precision when using a single review of a single record by an expert reviewer.^35^ Joint reviews using consensus to resolve disagreements result in only an illusory improvement in reliability.^36^ However, improvements in reliability can be achieved by averaging across multiple reviews.^37^ Our sample of 200 case record reviews per Trust, 2000 per epoch, 4000 case records in total and 4763 usable reviews is one of the largest reviews undertaken and produces adequate reliability (0.8–0.9) for distinguishing between Trusts. By examining the ‘difference-in-difference’ (comparisons of ratios), we have minimised confounding that would occur from comparisons between different Trusts, for example, from variation in case mix. We have previously shown that patients admitted as emergencies at weekends tend to be more severely ill than those admitted on weekdays.^8^ As severely ill patients may be more susceptible to healthcare error^38^ (mainly because the opportunity for error is greater in these patients),^39^ it might have been expected that error rates would be higher at weekends, but this is not the case.

## Conclusion

In summary, we find no evidence that in-hospital care is worse for patients admitted at weekends. We find improvements in hospital care between 2012–2013 and 2016–2017, manifest by a reduction in error rates and adverse events, better care processes and higher global quality ratings by the reviewers. It is possible that 7-day services has contributed to these improvements. Indications that community care performs less well at weekends, and has deteriorated between epochs, suggest that the causal pathways for the weekend effect extend into the prehospital setting. Policy makers should focus their efforts to improve acute and emergency care on a ‘whole system’ integrated approach, which focuses on all days of the week and includes care in the community.

## Data Availability

Data are available on reasonable request. We are willing to share our data for collaborative research.
