# Supplementary material for: Changes in weekend and weekday care quality of emergency medical admissions to 20 hospitals in England during implementation of the 7-day services national health policy
Source: BMJ Qual Saf. 2020 Oct 28;30(7):536–46. doi: 10.1136/bmjqs-2020-011165 (PMC8237174; doi:10.1136/bmjqs-2020-011165)
Supplement: Supplementary data [file bmjqs-2020-011165supp001.pdf]

APPENDIX 2 - ELECTRONIC SUPLEMENTARY MATERIAL (ESM)

ESM Table 1: Status of 20 participating Trusts in 2014

Trust	Size (number of beds, range)	Quintile (based on number of beds)	Specialist hours per 10 Emergency Admissions*
1	986-2037	**5**	40.2
2			34.1
3			15.5
4			10.7
5	787-984	**4**	38.9
6			36.1
7			18.5
8			15.4
9	636-783	**3**	50.7
10			35.7
11			11.4
12			8.1
13	479-627	**2**	47.0
14			38.0
15			9.6
16			7.0
17	240-470	**1**	38.6
18			34.7
19			11.1
20			8.8

*Adjusted using the formulae N x (1 / Response Rate) and rounded to the nearest integer

ESM Table 2: Characteristics of study population versus background population

	Case Notes Review data						All trusts/hositals data*					
	Total		W/E		W/D		Total		W/E		W/D	
	n	%	n	%	n	%	n	%	n	%	n	%
**Total**	4000		2000		2000		5818430		1411394		4407036	
**Age**												
Mean (SD)	61.1	22.310	61.7	22.379	60.5	22.271	61	22.350	61	22.780	61	22.190
Median (IQR)	65	43-81	66	44-81	64	42-80	65	43-80	66	43-81	65	43-80
**Gender**												
Male	1869	46.7	938	46.9	931	46.6	2639489	45.4	645166	45.7	1994323	45.3
Female	2131	53.3	1062	53.1	1069	53.5	3178941	54.6	766228	54.3	2412713	54.7
**Ethnicity**												
Caucasian	3140	78.5	1584	79.2	1556	77.8	4918775	84.5	1191464	84.4	3727311	84.6
Non_Caucasian	818	20.5	396	19.8	422	21.1	526384	9.0	128736	9.1	397648	9.0
Unknown	42	1.1	20	1.0	22	1.1	373271	6.4	91194	6.5	282077	6.4
**Top 5 primary discharge diagnosis (SHMI grouping)**												
Nonspecific chest pain	227	5.7	116	5.8	111	5.6	326876	5.6	72314	5.1	254562	5.8
Pneumonia (excluding TB/STD)	186	4.7	105	5.3	81	4.1	322014	5.5	85643	6.1	236371	5.4
Urinary tract infections	176	4.4	100	5.0	76	3.8	235612	4.0	64076	4.5	171536	3.9
Abdominal pain	156	3.9	70	3.5	86	4.3	243013	4.2	54697	3.9	188316	4.3
COPD & bronchiectasis	128	3.2	67	3.4	61	3.1	193168	3.3	48288	3.4	144880	3.3
**Other**	3127	78.2	1542	77.1	1585	79.3	4497747	77.3	1086376	77.0	3411371	77.4
**Length of stay**												
median (IQR)	2	0-5	2	0-5	1	0-4	1	0-5	2	0-5	1	0-5
**Zero length of stay**												
**ZLOS**	1107	27.7	512	25.6	595	29.8	1739129	29.9	388274	27.5	1350757	30.7
Not-ZLOS	2871	71.8	1477	73.9	1394	69.7	4078719	70.1	1023261	72.5	3054076	69.3
Unknown	22	0.6	11	0.6	11	0.6	0	0.0	0	0.0	0	0.0
**In-hospital mortality**												
Died	168	4.2	90	4.5	78	3.9	239719	4.1	63372	4.5	176281	4.0

*from Hospital Episode Statistics (HES), of those trusts participating in HiSLAC speciality intensity prevalence survey from 2014 to 2018 financial years

Bion J, *et al. BMJ Qual Saf* 2021; 30:536–546. doi: 10.1136/bmjqs-2020-011165

ESM Table 3a: Reviewers' assessment on pre-admission patient pathways

	BOTH EPOCHS						EPOCH 1						EPOCH 2					
	Total		W/E		W/D		Total		W/E		W/D		Total		W/E		W/D	
	n	%	n	%	n	%	n	%	n	%	n	%	n	%	n	%	n	%
**Total**	**4000**		**2000**		**2000**		**2000**		**1000**		**1000**		**2000**		**1000**		**1000**	
**Source of admission**																		
Own home	3389	84.7	1711	85.6	1678	83.9	1712	85.6	865	86.5	847	84.7	1677	83.9	846	84.6	831	83.1
Nursing or residential care home	257	6.4	121	6.1	136	6.8	127	6.4	60	6.0	67	6.7	130	6.5	61	6.1	69	6.9
No information available	247	6.2	114	5.7	133	6.7	105	5.3	46	4.6	59	5.9	142	7.1	68	6.8	74	7.4
Another hospital	30	0.8	16	0.8	14	0.7	12	0.6	6	0.6	6	0.6	18	0.9	10	1.0	8	0.8
No fixed abode	70	1.8	34	1.7	36	1.8	41	2.1	21	2.1	20	2.0	29	1.5	13	1.3	16	1.6
Missing	7	0.2	4	0.2	3	0.2	3	0.2	2	0.2	1	0.1	4	0.2	2	0.2	2	0.2
**Patient condition before admission**																		
Independent	2611	65.3	1292	64.6	1319	66.0	1333	66.7	667	66.7	666	66.6	1278	63.9	625	62.5	653	65.3
Needing help with some activities of daily living (ADLs)	672	16.8	332	16.6	340	17.0	351	17.6	169	16.9	182	18.2	321	16.1	163	16.3	158	15.8
Dependant on others for most/all ADLs including personal hygiene	384	9.6	219	11.0	165	8.3	174	8.7	100	10.0	74	7.4	210	10.5	119	11.9	91	9.1
Unable to determine; no relevant information in notes	325	8.1	152	7.6	173	8.7	139	7.0	62	6.2	77	7.7	186	9.3	90	9.0	96	9.6
Missing	8	0.2	5	0.3	3	0.2	3	0.2	2	0.2	1	0.1	5	0.3	3	0.3	2	0.2
**Referral to hospital mechanism**																		
999/ambulance transfer to ED	1873	46.8	1032	51.6	841	42.1	995	49.8	550	55.0	445	44.5	878	43.9	482	48.2	396	39.6
Self-presentation to ED (walk-in/own transport)	847	21.2	455	22.8	392	19.6	402	20.1	215	21.5	187	18.7	445	22.3	240	24.0	205	20.5
GP or deputising service referral (documented letter or phone call)	559	14.0	164	8.2	395	19.8	288	14.4	88	8.8	200	20.0	271	13.6	76	7.6	195	19.5
Unable to determine	553	13.8	282	14.1	271	13.6	236	11.8	123	12.3	113	11.3	317	15.9	159	15.9	158	15.8
Patient instructed by GP to attend ED, no formal evidence of referral	93	2.3	29	1.5	64	3.2	51	2.6	16	1.6	35	3.5	42	2.1	13	1.3	29	2.9
Urgent Care Centre or Walk-In Centre referral	45	1.1	26	1.3	19	1.0	16	0.8	4	0.4	12	1.2	29	1.5	22	2.2	7	0.7
Self-presentation, patient instructed to attend by 111 service	20	0.5	7	0.4	13	0.7	7	0.4	2	0.2	5	0.5	13	0.7	5	0.5	8	0.8
Missing	10	0.3	5	0.3	5	0.3	5	0.3	2	0.2	3	0.3	5	0.3	3	0.3	2	0.2
**Admission pathway**																		
ED / pre-admission area	3354	83.9	1757	87.9	1597	79.9	1664	83.2	870	87.0	794	79.4	1690	84.5	887	88.7	803	80.3
Direct admission to acute ward	451	11.3	160	8.0	291	14.6	247	12.4	93	9.3	154	15.4	204	10.2	67	6.7	137	13.7
Unable to determine	187	4.7	78	3.9	109	5.5	86	4.3	35	3.5	51	5.1	101	5.1	43	4.3	58	5.8
(blank)	8	0.2	5	0.3	3	0.2	3	0.2	2	0.2	1	0.1	5	0.3	3	0.3	2	0.2
**Pre_admission Vital Signs**																		
Full set + NEWS calculated	1765	44.1	914	45.7	851	42.6	761	38.1	395	39.5	366	36.6	1004	50.2	519	51.9	485	48.5
Full set, no NEWS calculated	1282	32.1	656	32.8	626	31.3	781	39.1	391	39.1	390	39.0	501	25.1	265	26.5	236	23.6
Incomplete vital signs	322	8.1	160	8.0	162	8.1	187	9.4	91	9.1	96	9.6	135	6.8	69	6.9	66	6.6
No vital signs documented	365	9.1	180	9.0	185	9.3	133	6.7	71	7.1	62	6.2	232	11.6	109	10.9	123	12.3
Not applicable: patient not admitted via ED: direct admission to ward	253	6.3	81	4.1	172	8.6	133	6.7	48	4.8	85	8.5	120	6.0	33	3.3	87	8.7
Missing	13	0.3	9	0.5	4	0.2	5	0.3	4	0.4	1	0.1	8	0.4	5	0.5	3	0.3

ESM Table 3b: Reviewers' assessments of post-admission pathways & care processes

	BOTH EPOCHS						EPOCH 1						EPOCH 2					
	Total		W/E		W/D		Total		W/E		W/D		Total		W/E		W/D	
	n	%	n	%	n	%	n	%	n	%	n	%	n	%	n	%	n	%
**Total**	**4000**		**2000**		**2000**		**2000**		**1000**		**1000**		**2000**		**1000**		**1000**	
**1st location post admission**																		
Acute Medical Unit (AMU/MAU)	1401	35.0	690	34.5	711	35.6	704	35.2	327	32.7	377	37.7	697	34.9	363	36.3	334	33.4
Unable to determine ward type	655	16.4	319	16.0	336	16.8	317	15.9	164	16.4	153	15.3	338	16.9	155	15.5	183	18.3
Clinical Decision Unit or short stay ward	659	16.5	356	17.8	303	15.2	353	17.7	187	18.7	166	16.6	306	15.3	169	16.9	137	13.7
Other (please specify):	422	10.6	196	9.8	226	11.3	200	10.0	103	10.3	97	9.7	222	11.1	93	9.3	129	12.9
General Surgery (including surgical assessment/operating theatre)	376	9.4	193	9.7	183	9.2	179	9.0	99	9.9	80	8.0	197	9.9	94	9.4	103	10.3
Medical sub-specialities including high care (eg: Coronary Care Unit, Renal Unit, Respiratory, Haematology, Oncology)	215	5.4	103	5.2	112	5.6	116	5.8	47	4.7	69	6.9	99	5.0	56	5.6	43	4.3
General medical ward	171	4.3	84	4.2	87	4.4	88	4.4	47	4.7	41	4.1	83	4.2	37	3.7	46	4.6
Older Peoples Medicine/Elderly Care Unit	46	1.2	27	1.4	19	1.0	19	1.0	11	1.1	8	0.8	27	1.4	16	1.6	11	1.1
Critical Care Unit / Intensive Care Unit (including High Dependency)	33	0.8	20	1.0	13	0.7	18	0.9	11	1.1	7	0.7	15	0.8	9	0.9	6	0.6
Missing	18	0.5	10	0.5	8	0.4	5	0.3	3	0.3	2	0.2	13	0.7	7	0.7	6	0.6
Rehabilitation	4	0.1	2	0.1	2	0.1	1	0.1	1	0.1	0	0.0	3	0.2	1	0.1	2	0.2
**Location appropriateness**																		
Yes, definitely appropriate	2469	61.7	1215	60.8	1254	62.7	1234	61.7	602	60.2	632	63.2	1235	61.8	613	61.3	622	62.2
Probably appropriate	854	21.4	443	22.2	411	20.6	455	22.8	235	23.5	220	22.0	399	20.0	208	20.8	191	19.1
Unable to determine	534	13.4	270	13.5	264	13.2	240	12.0	128	12.8	112	11.2	294	14.7	142	14.2	152	15.2
No	118	3.0	59	3.0	59	3.0	61	3.1	29	2.9	32	3.2	57	2.9	30	3.0	27	2.7
Missing	25	0.6	13	0.7	12	0.6	10	0.5	6	0.6	4	0.4	15	0.8	7	0.7	8	0.8
**Post_admission Vital Signs**																		
Full set + NEWS calculated	1940	48.5	1018	50.9	922	46.1	910	45.5	479	47.9	431	43.1	1030	51.5	539	53.9	491	49.1
Full set, no NEWS calculated	780	19.5	386	19.3	394	19.7	493	24.7	241	24.1	252	25.2	287	14.4	145	14.5	142	14.2
Incomplete vital signs	301	7.5	147	7.4	154	7.7	170	8.5	83	8.3	87	8.7	131	6.6	64	6.4	67	6.7
No vital signs documented	944	23.6	432	21.6	512	25.6	414	20.7	189	18.9	225	22.5	530	26.5	243	24.3	287	28.7
Missing	35	0.9	17	0.9	18	0.9	13	0.7	8	0.8	5	0.5	22	1.1	9	0.9	13	1.3
**Initial specialist review***																		
Specialist review documented in case record	1897	47.4	927	46.4	970	48.5	904	45.2	425	42.5	479	47.9	993	49.7	502	50.2	491	49.1
Probable specialist review but status of doctor uncertain	283	7.1	160	8.0	123	6.2	171	8.6	97	9.7	74	7.4	112	5.6	63	6.3	49	4.9
Unlikely that specialist review occurred	158	4.0	86	4.3	72	3.6	84	4.2	46	4.6	38	3.8	74	3.7	40	4.0	34	3.4
No evidence for specialist review documented	1248	31.2	639	32.0	609	30.5	585	29.3	319	31.9	266	26.6	663	33.2	320	32.0	343	34.3
Specialist review, time not documented, case record suggests > 14 hrs after admission	61	1.5	22	1.1	39	2.0	41	2.1	13	1.3	28	2.8	20	1.0	9	0.9	11	1.1
Specialist review, time not documented, but case record suggests < 14 hrs after admission	314	7.9	147	7.4	167	8.4	197	9.9	89	8.9	108	10.8	117	5.9	58	5.8	59	5.9
Specialist review documented < 14 hrs	1049	26.2	529	26.5	520	26.0	474	23.7	226	22.6	248	24.8	575	28.8	303	30.3	272	27.2
Probable specialist review documented < 14 hrs	140	3.5	78	3.9	62	3.1	78	3.9	47	4.7	31	3.1	62	3.1	31	3.1	31	3.1
Documented + probable specialist review < 14hrs	1189	29.7	607	30.4	582	29.1	552	27.6	273	27.3	279	27.9	637	31.9	334	33.4	303	30.3
Missing	39	1.0	19	1.0	20	1.0	18	0.9	11	1.1	7	0.7	21	1.1	8	0.8	13	1.3
**Palliative care discussed**																		
No, not required, patient appropriate for full treatment	2907	72.7	1425	71.3	1482	74.1	1471	73.6	718	71.8	753	75.3	1436	71.8	707	70.7	729	72.9
No, but would probably have been appropriate to consider some form of treatment limitation	437	10.9	217	10.9	220	11.0	252	12.6	131	13.1	121	12.1	185	9.3	86	8.6	99	9.9
Yes	275	6.9	163	8.2	112	5.6	112	5.6	70	7.0	42	4.2	163	8.2	93	9.3	70	7.0
No, but would definitely have been appropriate to limit treatment	239	6.0	124	6.2	115	5.8	129	6.5	63	6.3	66	6.6	110	5.5	61	6.1	49	4.9
DNACPR already in place prior to admission	110	2.8	55	2.8	55	2.8	22	1.1	10	1.0	12	1.2	88	4.4	45	4.5	43	4.3
**total above 3**	**624**	**15.6**	**342**	**17.1**	**282**	**14.1**	**263**	**13.2**	**143**	**14.3**	**120**	**12.0**	**361**	**18.1**	**199**	**19.9**	**162**	**16.2**
Missing	32	0.8	16	0.8	16	0.8	14	0.7	8	0.8	6	0.6	18	0.9	8	0.8	10	1.0
**Palliative care decision appropriateness (of those discussed)**																		
Yes, appropriate decision	273	99.3	162	99.4	111	99.1	111	99.1	70	100.0	41	97.6	162	99.4	92	98.9	70	100.0
Yes, but patient might have benefited from escalation	1	0.4	0	0.0	1	0.9	1	0.9	0	0.0	1	2.4	0	0.0	0	0.0	0	0.0
Missing	1	0.4	1	0.6	0	0.0	0	0.0	0	0.0	0	0.0	1	0.6	1	1.1	0	0.0
**Palliative care referral**																		
no	3844	96.1	1912	95.6	1932	96.6	1933	96.7	959	95.9	974	97.4	1911	95.6	953	95.3	958	95.8
yes	87	2.2	51	2.6	36	1.8	36	1.8	23	2.3	13	1.3	51	2.6	28	2.8	23	2.3
Missing	69	1.7	37	1.9	32	1.6	31	1.6	18	1.8	13	1.3	38	1.9	19	1.9	19	1.9
**Admission avoidable**																		
no	2919	73.0	1498	74.9	1421	71.1	1475	73.8	749	74.9	726	72.6	1444	72.2	749	74.9	695	69.5
possibly	783	19.6	361	18.1	422	21.1	388	19.4	184	18.4	204	20.4	395	19.8	177	17.7	218	21.8
yes	269	6.7	124	6.2	145	7.3	127	6.4	59	5.9	68	6.8	142	7.1	65	6.5	77	7.7
Missing	29	0.7	17	0.9	12	0.6	10	0.5	8	0.8	2	0.2	19	1.0	9	0.9	10	1.0

*Categories in this section are not mutually exclusive

Bion J, *et al. BMJ Qual Saf* 2021; 30:536–546. doi: 10.1136/bmjqs-2020-011165

ESM Table 4: Reviewers' assessment on error, adverse events, and global quality of care

	BOTH EPOCHS						EPOCH 1						EPOCH 2					
	Total		W/E		W/D		Total		W/E		W/D		Total		W/E		W/D	
	n	%	n	%	n	%	n	%	n	%	n	%	n	%	n	%	n	%
**Total**	**4000**		**2000**		**2000**		**2000**		**1000**		**1000**		**2000**		**1000**		**1000**	
**Number of errors**																		
No error	2970	74.3	1486	74.3	1484	74.2	1447	72.4	723	72.3	724	72.4	1523	76.2	763	76.3	760	76.0
1	645	16.1	328	16.4	317	15.9	338	16.9	178	17.8	160	16.0	307	15.4	150	15.0	157	15.7
2	208	5.2	100	5.0	108	5.4	121	6.1	53	5.3	68	6.8	87	4.4	47	4.7	40	4.0
3	83	2.1	36	1.8	47	2.4	43	2.2	18	1.8	25	2.5	40	2.0	18	1.8	22	2.2
4	31	0.8	17	0.9	14	0.7	22	1.1	12	1.2	10	1.0	9	0.5	5	0.5	4	0.4
5	13	0.3	9	0.5	4	0.2	7	0.4	5	0.5	2	0.2	6	0.3	4	0.4	2	0.2
6	9	0.2	4	0.2	5	0.3	4	0.2	1	0.1	3	0.3	5	0.3	3	0.3	2	0.2
7	3	0.1	1	0.1	2	0.1	2	0.1	0	0.0	2	0.2	1	0.1	1	0.1	0	0.0
8	2	0.1	1	0.1	1	0.1	2	0.1	1	0.1	1	0.1	0	0.0	0	0.0	0	0.0
13	1	0.0	0	0.0	1	0.1	1	0.1	0	0.0	1	0.1	0	0.0	0	0.0	0	0.0
15	1	0.0	1	0.1	0	0.0	1	0.1	1	0.1	0	0.0	0	0.0	0	0.0	0	0.0
Missing	34	0.9	17	0.9	17	0.9	12	0.6	8	0.8	4	0.4	22	1.1	9	0.9	13	1.3
**Error in care**																		
**Yes**	996	24.9	497	24.9	499	25.0	541	27.1	269	26.9	272	27.2	455	22.8	228	22.8	227	22.7
**No**	2970	74.3	1486	74.3	1484	74.2	1447	72.4	723	72.3	724	72.4	1523	76.2	763	76.3	760	76.0
**Missing**	34	0.9	17	0.9	17	0.9	12	0.6	8	0.8	4	0.4	22	1.1	9	0.9	13	1.3
**Total number of errors**																		
	1618		803		815		914		440		474		704		363		341	
**Mean number of errors per patient admission** (total number of errors / number of patient admission excluding errors unknown)																		
	0.408		0.405		0.41		0.46		0.444		0.476		0.36		0.37		0.35	
**Location of errors***	1617		802		815		914		440		474		703		362		341	
1, Outside hospital (primary care , ambulance etc)	31	1.9	11	1.4	20	2.5	16	1.8	7	1.6	9	1.9	15	2.1	4	1.1	11	3.2
2, In the ED or linked area before admission	554	34.3	298	37.2	256	31.4	310	33.9	167	38.0	143	30.2	244	34.7	131	36.2	113	33.1
3, AMU or equivalent area	554	34.3	255	31.8	299	36.7	312	34.1	134	30.5	178	37.6	242	34.4	121	33.4	121	35.5
4, Acute ward (other than AMU)	294	18.2	158	19.7	136	16.7	170	18.6	87	19.8	83	17.5	124	17.6	71	19.6	53	15.5
5, Speciality (ICU / HDU, coronary care, renal, respiratory, elderly care, rehab)	174	10.8	74	9.2	100	12.3	102	11.2	42	9.5	60	12.7	72	10.2	32	8.8	40	11.7
6, Diagnostic area, radiology	10	0.6	6	0.7	4	0.5	4	0.4	3	0.7	1	0.2	6	0.9	3	0.8	3	0.9
**Error typology**	2409		1208		1201		1391		681		710		1018		527		491	
1, Assessment	768	31.9	379	31.4	389	32.4	446	32.1	214	31.4	232	32.7	322	31.6	165	31.3	157	32.0
2, Medication	317	13.2	157	13.0	160	13.3	191	13.7	98	14.4	93	13.1	126	12.4	59	11.2	67	13.6
3, Treatment and management	702	29.1	346	28.6	356	29.6	406	29.2	193	28.3	213	30.0	296	29.1	153	29.0	143	29.1
4, Infection control	18	0.7	8	0.7	10	0.8	5	0.4	2	0.3	3	0.4	13	1.3	6	1.1	7	1.4
5, Invasive procedures	25	1.0	15	1.2	10	0.8	17	1.2	10	1.5	7	1.0	8	0.8	5	0.9	3	0.6
6, Monitoring	114	4.7	57	4.7	57	4.7	64	4.6	33	4.8	31	4.4	50	4.9	24	4.6	26	5.3
7, Resuscitation	29	1.2	22	1.8	7	0.6	16	1.2	12	1.8	4	0.6	13	1.3	10	1.9	3	0.6
8, Communication	369	15.3	188	15.6	181	15.1	202	14.5	96	14.1	106	14.9	167	16.4	92	17.5	75	15.3
9, Other	67	2.8	36	3.0	31	2.6	44	3.2	23	3.4	21	3.0	23	2.3	13	2.5	10	2.0
**Mean number of error typology of each category per patient admission**	0.607		0.609		0.606		0.700		0.686		0.713		0.515		0.532		0.497	
1, Assessment	0.194		0.191		0.196		0.224		0.216		0.233		0.16		0.17		0.16	
2, Medication	0.080		0.079		0.081		0.096		0.099		0.093		0.06		0.06		0.07	
3, Treatment and management	0.177		0.174		0.180		0.204		0.195		0.214		0.15		0.15		0.14	
4, Infection control	0.005		0.004		0.005		0.003		0.002		0.003		0.01		0.01		0.01	
5, Invasive procedures	0.006		0.008		0.005		0.009		0.01		0.007		0		0.01		0	
6, Monitoring	0.029		0.029		0.029		0.032		0.033		0.031		0.03		0.02		0.03	
7, Resuscitation	0.007		0.011		0.004		0.008		0.012		0.004		0.01		0.01		0	
8, Communication	0.093		0.095		0.091		0.102		0.097		0.106		0.08		0.09		0.08	
9, Other	0.017		0.018		0.016		0.022		0.023		0.021		0.01		0.01		0.010	
**Error associated with adverse event**																		
Yes	128	7.9	58	7.2	70	8.6	91	10.0	39	8.9	52	11.0	37	5.3	19	5.2	18	5.3
No	757	46.8	370	46.1	387	47.5	405	44.3	192	43.6	213	44.9	352	50.0	178	49.0	174	51.0
Insufficient evidence	733	45.3	375	46.7	358	43.9	418	45.7	209	47.5	209	44.1	315	44.7	166	45.7	149	43.7
**Preventability of adverse event (of those associated with adverse event)**																		
1, Virtually no evidence for preventability	0	0.0	0	0.0	0	0.0	0	0.0	0	0.0	0	0.0	0	0.0	0	0.0	0	0.0
2, Slight to modest evidence of preventability	16	12.5	11	8.6	5	3.9	16	12.5	11	8.6	5	3.9	0	0.0	0	0.0	0	0.0
1&2	16	12.5	11	8.6	5	3.9	16	12.5	11	8.6	5	3.9	0	0.0	0	0.0	0	0.0
3, Possibly preventable, but not very likely (less than 50-50, but close call)	21	16.4	7	5.5	14	10.9	12	9.4	3	2.3	9	7.0	9	7.0	4	3.1	5	3.9
4, Probably preventable (more than 50-50, but a close call)	26	20.3	12	9.4	14	10.9	21	16.4	9	7.0	12	9.4	5	3.9	3	2.3	2	1.6
3 & 4	47	36.7	19	14.8	28	21.9	33	25.8	12	9.4	21	16.4	14	10.9	7	5.5	7	5.5
5, Strong evidence for preventability	41	32.0	14	10.9	27	21.1	28	21.9	9	7.0	19	14.8	13	10.2	5	3.9	8	6.3
6, Virtually certain evidence of preventability	24	18.8	14	10.9	10	7.8	14	10.9	7	5.5	7	5.5	10	7.8	7	5.5	3	2.3
5 & 6	65	50.8	28	21.9	37	28.9	42	32.8	16	12.5	26	20.3	23	18.0	12	9.4	11	8.6
**Global assessment**																		
1, Completely	1579	39.5	778	38.9	801	40.1	751	37.6	366	36.6	385	38.5	828	41.4	412	41.2	416	41.6
2, Substantially	1659	41.5	846	42.3	813	40.7	837	41.9	435	43.5	402	40.2	822	41.1	411	41.1	411	41.1
1 & 2	3238	81.0	1624	81.2	1614	80.7	1588	79.4	801	80.1	787	78.7	1650	82.5	823	82.3	827	82.7
3, Partially	623	15.6	303	15.2	320	16.0	333	16.7	159	15.9	174	17.4	290	14.5	144	14.4	146	14.6
4, Very little	83	2.1	43	2.2	40	2.0	56	2.8	27	2.7	29	2.9	27	1.4	16	1.6	11	1.1
5, Not at all	23	0.6	12	0.6	11	0.6	8	0.4	3	0.3	5	0.5	15	0.8	9	0.9	6	0.6
4 & 5	106	2.7	55	2.8	51	2.6	64	3.2	30	3.0	34	3.4	42	2.1	25	2.5	17	1.7
Missing	33	0.8	18	0.9	15	0.8	15	0.8	10	1.0	5	0.5	18	0.9	8	0.8	10	1.0

*one error had no information on location

Bion J, *et al. BMJ Qual Saf* 2021; 30:536–546. doi: 10.1136/bmjqs-2020-011165

ESM Figure 1: Mean number of errors identified per case notes by reviewer

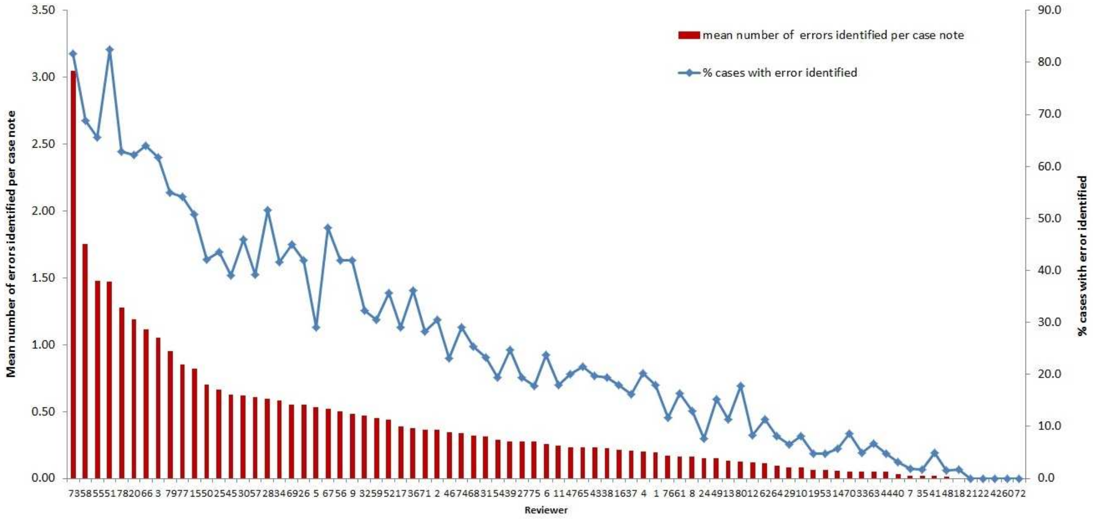

Bion J, *et al. BMJ Qual Saf* 2021; 30:536–546. doi: 10.1136/bmjqs-2020-011165
